# Ellagitannins and Other Polyphenols Along with Dietary Components of the Rosaceae Medicinal Plants

**DOI:** 10.3390/molecules30234574

**Published:** 2025-11-27

**Authors:** Monika Kosmala, Joanna Milala, Elżbieta Karlińska

**Affiliations:** Institute of Food Technology and Analysis, Lodz University of Technology, Stefanowskiego 2/22, 90-537 Lodz, Polandelzbieta.karlinska@p.lodz.pl (E.K.)

**Keywords:** *Sanguisorba officinalis* L., *Geum urbanum* L., *Agrimonia procera* Wallr., ellagitannins, dietary fiber

## Abstract

The edible medicinal plants *Sanguisorba officinalis* L. (great burnet), *Geum urbanum* L. (wood avens), and *Agrimonia procera* Wallr. (fragrant agrimony) of the Rosaceae family are a several times richer source of tannins, especially ellagitannins, than berries containing 3.0, 2.1, and 3.4 g/100 g phenolics in fresh matter. The herbs are traditionally used as anti-bacterial, anti-diarrheal, and anti-inflammatory agents for the intestines. As a source of phenolics, mostly ellagitannins, the herbs have a potentially beneficial effect on the lipid profile of the blood by reducing total cholesterol, LDL-cholesterol, and triglycerides. They are also a good source of dietary fiber (6.5 for *Sanguisorba*, 8.2 for *Geum*, and 11.1 g/100 g fresh matter for *Agrimonia*) and vitamin C, all 0.1 g/100 g fresh matter. Due to their resistance to fungal diseases and pathogens, the medicinal plants are free from pesticide residues. *Sanguisorba*, *Geum*, and *Agrimonia* are tasty and aromatic and can be the basis of dishes, salads, or beverages.

## 1. Introduction

Nutritional studies show that regular fruit and vegetable intake can prevent the development of coronary heart disease [1,2,3]. There is also moderate evidence that adequate nutrition has an impact on reducing the incidence of type 2 diabetes, some types of cancer [4,5,6,7], as well as overweight and obesity [8]. The microstructure of food plays a major role in the prevention and therapy of some chronic human diseases, as it determines the bioavailability of active ingredients, including their release from the intestines into the bloodstream [9]. The results of clinical trials and long-term observational studies show that the nutritional importance of fruits and vegetables is much larger than previously thought. The recommendations of dieticians are reflected in the latest healthy eating pyramid, where physical activity appears to be the most important factor for maintaining health. The need to change the diet to a balanced, healthier one entails searching for dishes with an increased share of ingredients considered to be beneficial for health. The increased number of vegetarians and a great interest in nutrition based primarily on plant food is also an important issue. New flavors and sources of nutrients rich in bioactive substances are sought after. Wild-growing and cultivated medicinal plants (herbs or spices) can meet these needs. Often, such perennial plants are characterized by high resistance to diseases and are free from plant protection product residues. Therefore, they have attributes that are appropriate for safe and ecological products. Herbs, fresh or dried, due to the presence of physiologically active substances, affect human metabolism and can be used for medicinal purposes, and some also as spices and ingredients of dishes. Edible herbs can form the basis of new dishes and enrich the taste and nutritional value of dishes already known [10]. Plants of the Rosaceae family, especially the burnet, agrimony, and avens, are used in medicine as anti-bacterial, anti-diarrheal, and anti-inflammatory herbs for the intestines. Their actions are related to the presence of a group of polyphenol compounds, called tannins, which are divided into hydrolysable and condensed tannins. The first includes ellagitannins (ETs) and the second includes proanthocyanidins (PCs). ETs are esters, usually β-D-glucose and hexahydroxydiphenic acid (HHDP), and can be divided into simple ETs, C-glycosidic ETs, complex tannins (condensates of C-glycosidic tannins with flavan-3-ol), and oligomers up to pentamers [11]. One of the features of ETs is their ability to hydrolyze in an acidic environment. The product of the hydrolysis of ETs in the gastrointestinal tract is ellagic acid, which is metabolized via the colonic microbiota to the derivatives of hydroxy-6H-dibenzopyran-6-one, called urolithins. The importance of ET metabolites is not fully understood. The results of the research show their antioxidant effect [12], anti-inflammatory properties [13,14], and anti-estrogen/estrogen activity because of their structural analogy to estrogens [15]. ETs with their metabolites, urolithins, have been found to express potential against digestive diseases, such as irritable bowel syndrome, peptic ulcers, gastritis, colon cancer, esophageal cancer, and pancreatic cancer [7]. As a result, there is an increasing demand for the commercialization of nutraceuticals and dietary supplements containing ETs. However, there is still a lack of specific regulations to ensure the quality, efficacy, safety, and traceability of these products. The development and implementation of robust regulatory standards and guidelines are a top priority to protect consumers and ensure the high quality of these products [7].

Herbs from the Rosaceae family, such as *Sanguisorba officinalis*, *Agrimonia procera*, and *Geum urbanum*, are a valuable, though still insufficiently studied, source of bioactive compounds. The selection of these three species was dictated by their shared taxonomic origin and similar chemical profile, which includes the presence of tannins, phenolic acids, flavonoids, and ETs. However, as indicated by available literature data, the quantitative and qualitative polyphenol composition of these plants varies [16,17,18,19,20,21,22,23,24], making them an interesting subject for comparative research. An analysis of the nutritional and polyphenol profiles of *Sanguisorba officinalis*, *Agrimonia procera*, and *Geum urbanum* may provide new insights into the potential of these raw materials as components of herbal preparations, dietary supplements, or functional foods.

Existing research proves that the herbs are non-toxic. *Sanguisorba* is an edible plant considered safe for food in many parts of the world, and its industrial use is significant. The plant has many valuable biological properties, such as anti-cancer, antioxidative, antimicrobial, antiviral, anti-Alzheimer’s disease, and anti-inflammatory activity [16]. The lack of toxicity and strong biological activity of *Sanguisorba* spp. has already been proven, and this confirms that the plant can be a material for obtaining supplements [16]. Fragrant agrimony is a pharmacopeial plant material, being a source of ETs—agrimoniin [17]. It was found that *Geum urbanum* L. extract, containing ellagic acid, ETs, and other compounds, exhibited antiviral activity, promising anti-cancer effects, and, importantly, is characterized by favorable toxicological potential both in vitro and in vivo in H-albino mice, showing no signs of toxicity or pathological changes in the liver and kidneys after two weeks of oral administration [18].

Although *Sanguisorba officinalis*, *Agrimonia procera*, and *Geum urbanum* have been previously investigated, comparative studies focusing simultaneously on their nutritional composition and polyphenolic profile are still scarce. Most available reports describe individual species or selected compound groups, often under non-comparable experimental conditions. Our study provides a systematic comparative evaluation of these three *Rosaceae* species, highlighting both quantitative and qualitative differences in their polyphenolic composition, and nutritional value.

The aim of the research was to prove that medicinal plants in the early stage of development are several times richer sources of ET than berries. As they are resistant to fungal diseases and pathogens, the medicinal plants are free from pesticides, and that is why the herbs can be the basis of tasty dishes, beverages, and salads, not only in the form of infusions or decoctions. As the herbs can be consumed directly, the nutritional as well as detailed phenolic composition of the edible medicinal plants *Sanguisorba officinalis* L. (great burnet), *Geum urbanum* L. (wood avens), and *Agrimonia procera* Wallr. (fragrant agrimony) was the aim of this research. We have also proven that these herbs are a good source of dietary fiber, which is a vital component of a healthy digestive system.

## 2. Results

**Figure 1:**
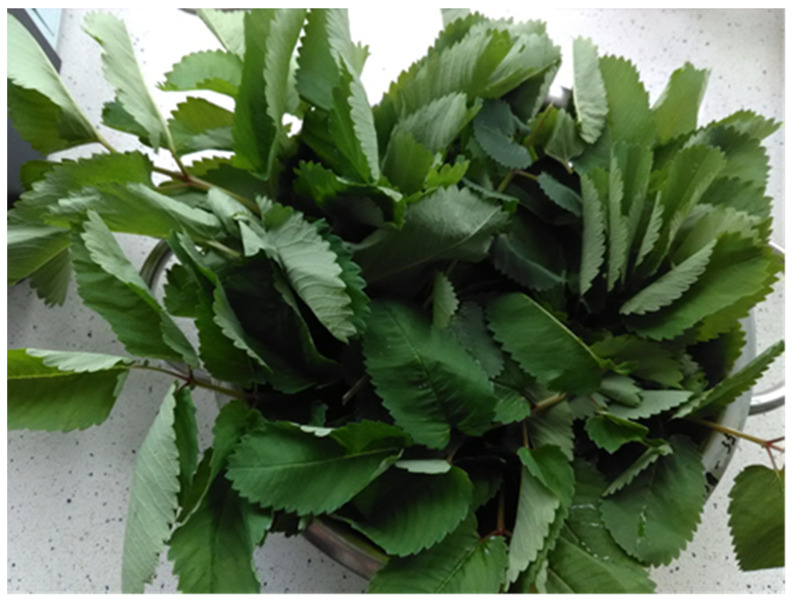
*Sanguisorba officinalis* L., great burnet.

**Figure 2:**
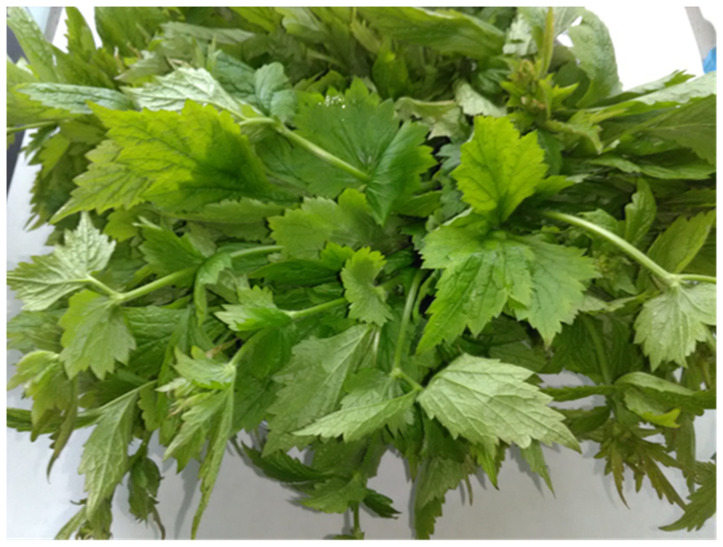
*Geum urbanum* L., wood avens.

**Figure 3:**
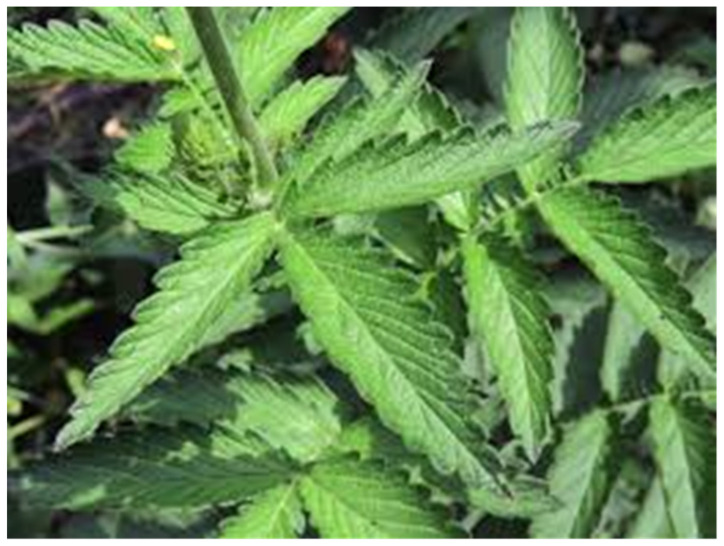
*Agrimonia procera* Wallr, fragrant agrimony.

Among the substances found in herbs, proteins and nitrogen compounds, carbohydrates, fats, glycosides, alkaloids, tannins, flavonoids, organic acids, vitamins, and minerals should be mentioned [10]. Fresh leaves of the herbs, great burnet (Figure 1), wood avens (Figure 2), and fragrant agrimony (Figure 3), contain mainly water (dry weight: avens, 18.7, great burnet, 19.7, and fragrant agrimony, 24 g/100 g) (Table 1).

The herbs are a good source of total dietary fiber (TDF) (burnet, 6.5 g/100 g, avens, 8 g/100 g, and agrimony, 11.1 g/100 g), mainly the insoluble fraction (IDF), while soluble dietary fiber (SDF) was below 1 g/100 g. Such high fiber content is comparable to levels typical for legumes (green peas 6 g/100 g, dried peas 15 g/100 g, dried white beans 15.7 g/100 g, and green beans 3.9 g/100 g [25]. Among typical fruits and vegetables, only raspberry (6.7 g/100 g), black currant (7.9 g/100 g), and horseradish (7.3 g/100 g) [25] have fiber content like the discussed herbs. Protein was 3–4 g/100 g. Ash content was about 2 g/100 g. The metabolized carbohydrates are 3.0–3.8 g/100. The content of vitamin C in fresh herbs *Geum urbanum* L., fragrant agrimony *Agrimonia procera* Wallr., and *Sanguisorba officinalis* L. was 0.1 g/100 g. The vitamin C content in fresh herbs is comparable to the values for strawberry fruit (66 mg/100 g), spinach (68 mg/100 g), and parsley (178 mg/100 g) [25]. Ceccanti et al. [26] showed that wild and domesticated *Sanguisorba minor* Scop. plants contain, per 100 g of dry matter, appropriately, moisture 75.02 and 71.20, ash 7.80 and 10.10, proteins 18.80 and 23.10, fat 2.72 and 4.00, and carbohydrates 70.70 and 62.79.

Karlińska et al. [22] also found that the aboveground parts of fragrant agrimony, especially the leaves, were characterized by a high content of protein and dietary fiber, with a relatively low-fat content. In the leaves of fragrant agrimony, the contents of protein and dietary fiber amounted to 12% and 55% DM, whereas in the stems, they amounted to 7% and 75% DM [22]. Other researchers also found that herb plants *Sanguisorba*, *Geum*, and *Agrimonia* of the Rosaceae family are rich in vitamin C and also polyphenols, especially ellagitannins [21,22,23,24,25,26,27,28,29]. The polyphenol content in the fresh herbs ranged from 2.1 to 3.4 g/100 g fresh matter. Similar results were obtained by Karlińska et al. [22] for fragrant agrimony leaves—10.5% of polyphenols DM, while DM was 30.45%. Analyzed herbs from the Rosaceae family are mainly the source of ETs (Table 2) [17,18,19,20,21,22].

In the case of burnet, 86% of polyphenols are oligomeric ellagitannins (sanguiin H6 and lambertianin C) (Table 3). Other polyphenols include quercetin glycosides, flavan-3-ols, monomeric ellagitannins, and chlorogenic acids. Wood avens is a source of dimeric gemin A, which accounts for 72% of the total polyphenols, 8% of chlorogenic acids, 8% of monomeric ellagitannin, flavan-3-ols in a smaller amount, and quercetin glycosides in trace amounts (Table 4). In the case of agrimony, 41% of the total polyphenols are dimeric agrimoniin, 28% polyphenols of flavan-3-ols, the rest are rutin, quercetin, and luteolin glycosides, and tiliroside (Table 5). Fresh herbs of burnet and avens contain negligible amounts of flavan-3-ols compared to agrimony (0.95% in fresh weight) (Table 2).

## 3. Discussion

Plants of the genera, great burnet *Sanguisorba*, fragrant agrimony *Agrimonia,* and wood avens *Geum,* are also classified as edible plants and spices. The perennials are characterized by high frost and disease resistance. In addition to frost and disease resistance and high biomass productivity, plants of the genera *Sanguisorba* (*S. officinalis*, *S. minor*), *Geum* (*G. urbanum*, *G. rivale*), and *Agrimonia* (*A. procera*) are characterized by high content of ellagitannins. The burnet belonging to the genus *Sanguisorba* species *S. officinalis* L. (great burnet) is a perennial with a strong, thick rhizome, leaf rubella, and odd-stemmed stems up to 1 m high and ends with heads of tiny dark purple flowers [27]. Burnet blooms in the period from June to August. The fruit is an achene. In Poland, the plant is widespread on lowlands and pastures, and in the lower parts of the Tatra Mountains. The herbal raw material is a herb of great burnet—*Herba Sanguisorbae* and a great burnet rhizome—*Radix Sanguisorbae*. The herb is harvested at the beginning of flowering, while the rhizomes are harvested in autumn or in spring. After carefully cleaning, the rhizomes are cut into pieces and dried under natural conditions or in a dryer at a temperature of up to 60 °C. The herb, after milling, is stored in a dry and dark place. The great burnet rhizomes contain about 20% tannins, including ellagitannins, 2–4% triterpene saponins, flavonoids (rutin and kaempferol), phenolic acids, vitamin C and K (phytochinones), phytosterols, and phytoncides. Herbs with flowers contain less tannins; however, they additionally contain cyanidin glycosides (cyanidin-3-glucoside and cyanidin-3,5-diglucoside) as well as bitterness, sugars, ethereal oil, and quercetin. The ingredients of great burnet rhizomes and herb have anti-inflammatory, astringent, anti-exudative, anti-diarrheal, anti-edema, anti-hemorrhagic, inhibit menstrual bleeding, diuretic, antiseptic, antiulcer, and anti-seborrheic properties. Tannins have astringent, slightly disinfecting, and anti-hemorrhagic properties. Great burnet rhizomes and herbs used to be a popular tannin drug used for stopping bleeding. Stimulating milk production properties are additionally attributed to the herb. In some countries, young leaves are eaten raw like lettuce or prepared like spinach. The related species is small burnet *S. minor* [34]. The possibilities of its cultivation and its antioxidant activity have been described in [28]. In the article, it was shown that, among the two forms, Dutch has a lower leaf width compared to the German one, and the German form showed a higher content of vitamin C and total polyphenols determined by Folin–Ciocalteu’s method calculated as gallic acid. Small burnet is easy to grow from seeds to be used as a herbal raw material and as a vegetable. For this purpose, young leaves suitable for freezing are collected. There is no exact data on the polyphenolic composition of this species of small burnet, which additionally forms hybrids (forms, populations). Both small burnet *S. minor* and narrow-leaf burnet *S. tenuifolia* are related to great burnet [35]. Taking into account their usefulness as a herbal raw material, they are less valuable. Narrow-leaf burnet *S. tanuifolia* and Japanese burnet *S. obtusa* are widespread as long-blooming ornamental plants, giving numerous varieties with colorful flowers and nicely formed leaves. The composition and nutritional use of these species are unknown. *G. urbanum* L. (wood avens), belonging to the genus *Geum*, is a large rhizome perennial with many light brown fibrous roots, a stem height up to 1 m, and leaves placed alternately wedge-shaped [35]. The leaves are covered with spreading hairs, and their margins are toothed. The small yellow flowers with five roundish, spreading petals are placed on solitary, terminal stalks. Flowering period falls in June–August. It occurs commonly on wet shaded meadows, undergrowth, forests, and orchards. It has been known since antiquity due to its antitussive effect. A herbal raw material is avens rhizomes, *Rhizoma Gei urbani*, and, less frequently, leaves from *Folium Gei urbani*. Rhizomes are collected in the autumn in October–November or in spring, in March. After thorough washing and removal of green parts, they are dried at a maximum temperature of 35 °C. Avens rhizomes contain about 20% of tannins, including ellagitannins (gemin A), gein (eugenol glycoside), bitterness, flavonoids, phenolic acids, organic acids, and essential oils of 0.1%. The active components of avens rhizomes show strong astringent and choleretic as well as bactericidal, anti-inflammatory, and anti-cancer properties, and avens rhizomes are also used to treat the effects of chemotherapy. Avens stimulates the secretion of gastric juices and removes unpleasant smells from the mouth. Preparations (extracts, decoctions, and intracts) from avens rhizomes are used internally in diseases of the gastrointestinal tract. Decoctions and intractions (from fresh raw materials) are used externally in flu and angina conditions, in wounds difficult to heal, in mouth rinsing, seborrhea, dermatitis, and in fungal infections by *Candida*. Usually, the water avens species *G. rivale* L. is considered to be an equivalent herbal raw material. The plant reaches a height of up to 70 cm. The species occurs in alluvial forests and in wet meadows, in shaded positions. This avens has characteristic bell-shaped, overhanging flowers, growing on red-brown, long stems. The flowers are red-brown on the outside and yellow inside. Garden varieties with different colors are also known [35]. Agrimony belongs to the genus *Agrimonia*, *A. eupatoria* species (common agrimony, hepatica herb) [34]. It is a perennial plant with a short rhizome, rosette leaf, pinnate, and a straight stem, growing up to 1 m with leaves alternating. The stem ends with a loose ear with five-colored golden-yellow flowers. Agrimony blooms from June to September. Its fruit is an achene. In the natural environment, agrimony occurs mainly on dry meadows, pastures, forest glades, and on the edges of forests. Apart from common agrimony, there is also fragrant agrimony, *A. procera* Wallr. and hairy agrimony *A. pilosa* Ledeb. also obtained as a herbal raw material. A herbal raw material is agrimony herb—*Herba Agrimoniae*. The herb is harvested at the beginning of flowering, then the herb is dried under natural conditions in a shaded and well-ventilated place or in dryers at a temperature not exceeding 60 °C. The dried herb has a pleasant aroma and slightly bitter taste. Agrimony herb contains from 3 to 5% of tannins, flavonoids (quercetin, kaempferol, luteolin, apigenin), triterpene (ursolic acid), choline, phytosterols, and silica, as well as 0.2% of essential oils. Flavonoids and essential oils increase the production and flow of bile from the liver to the gall bladder and duodenum. Tannins and flavonoids inhibit inflammation and exudates in the gastrointestinal mucosa. Tannins have slightly astringent and antiseptic properties. Together, the components have confirmed antibacterial and anti-dandruff properties, and they inhibit excessive fermentation and putrefaction in the gut. Formerly, preparations of agrimony herb were recommended in the treatment of cholelithiasis and bile stasis. Agrimony was recommended even in jaundice. Due to the high content of tannins, agrimony herb is used to treat diarrhea and to rinse the mouth and throat in case of inflammation. Antiseptic and anti-inflammatory infusions from *A. eupatoria* are recommended to occupational voice users [36]. Agrimony also belongs to wild edible plants. In pharmacy, under the term tannins, a large group of polyphenol compounds, easily soluble in water, that form persistent combinations with proteins, alkaloids, and heavy metals, is meant. Tannins are divided into hydrolyzing tannins and condensed tannins. Their therapeutic value is based on astringent properties (they coagulate proteins of the mucous membrane and tissues), and, as a result, irritation and pain are reduced; they also inhibit minor bleeding. Decoctions of herbs rich in tannins (blackberry leaf, agrimony herb, burnet root, and others) are most often used externally against inflammation of the mouth, catarrhal mucosa, bronchitis, and local bleeding.

Appropriate analytical techniques and methods in accordance with the applicable standards and requirements provide an evaluation of the composition and quality of raw materials and medicinal preparations. In the case of the determination of certain groups of active substances, e.g., tannins, simple but not specific traditional methods are used, which are slowly supported by sophisticated instrumental methods. The phytochemical requirements for *Herba Agrimoniae* are contained in the European Pharmacopoeia [37] using the traditional skin powder method. According to this method, agrimony should contain not less than 2% of total tannin content (TTC). Phytochemical and chromatographic research [17] showed that common agrimony contains from 0.2 to 0.7% of agrimoniin, determined by HPLC. In turn, fragrant agrimony *A. procera* showed a TTC content of 2.7–3.5% m/m, and the agrimoniin content determined by HPLC was 2.1–3.7% m/m. This indicates that fragrant agrimony herb is a guarantor of the proper content of TTC, and may also be considered as a satisfactory source of agrimoniin and as an alternative raw material to dried strawberry leaves (*Fragariae folium*), in which the TTC content is about 1.0% m/m [27], and silverweed herb *Anserinae herba*, in which TTC content is 1.5% m/m. Plants of the genus *Agrimonia* L., the common agrimony *Agrimonia eupatoria* L., and the fragrant agrimony *Agrimonia procera* Wallr. (synonym *Agrimonia odorata* Mill.) are sources of both ellagitannins and flavonoids [23]. The polyphenolic content of *A. procera* was found to be 3.9%, 3.2%, 2.9%, 1.8%, and 1.1%, and that of *A. eupatoria* was determined to be 1.3%, 0.3%, 0.9%, 0.6%, and 0.5% in the dry matter of leaves, stems, fruits, seeds, and hypanthia, respectively. Agrimoniin was the main polyphenolic compound in the aerial parts of the studied Agrimonia species, except for *A. procera* hypanthia. Both plants are also a valuable source of flavonoid glycosides, especially apigenin, luteolin, and quercetin [23].

Karlińska et al. [22] found that ellagitannins and flavan-3-ols are the dominant polyphenols of all morphological parts of fragrant agrimony. The ET content, calculated on dry matter, was almost 5% in leaves and stems, 8% in roots, and 16% in underground buds, with the agrimoniin accounting for about 98% of the determined sum of ETs. Flavan-3-ols, calculated on dry matter, were present in fragrant agrimony in amounts of almost 3.7% in leaves, over 2% in stems and underground buds, and 4.6% in roots. Flavonols and flavones in fragrant agrimony were present in total amounts not exceeding 2% of the dry mass of leaves, 1.5% of the mass of underground buds, and slightly more than 0.1% of the mass of stems. In our research, similar results were obtained.

The research by Lachowicz et al. [19] on the content and profile of polyphenols in the leaves, flowers, roots, and stalks of the medicinal plant *Sanguisorba officinalis* L. revealed the presence of 130 phenolic compounds, with hydrolysable tannins constituting the dominant group. Flowers were the richest source of phenolic compounds, while stalks were the poorest. The leaves had the highest antiradical activity. The morphological parts studied contained tannins, flavan-3-ols, phenolic acids, flavonols, and anthocyanins in the case of flowers. Among the ellagitannins were pedunculagin and its isomers, tellimagrandin I, castalagin/vescalagin isomer, lambertianin C, galloyl-bis-HHDP-glucose (potentilin/casuarictin isomer), as well as ellagic acid derivatives and others. Among the flavonols, the presence of quercetin and kaempferol glycosides was found. In addition, the authors indicate that flowers and leaves have α-amylase, α-glucosidase, and pancreatic lipase, as well as antiproliferative activity, reflected in the inhibition of the survival of cancer cells of pancreatic duct adenocarcinoma, colon adenocarcinoma, and bladder cancer, as well as T-cell leukemia cells.

The *Geum aleppicum* Jacq. is used in many traditional medical systems to treat diabetes. Kashchenko et al. [33] identified in *Geum aleppicum* ellagic acid derivatives, ETs, flavonoids, and triterpenoids. Gemin A, miquelianin, nigaichigoside F1, and 3,4 dihydroxybenzoic acid 4-O-glucoside were the dominant compounds in the *G. aleppicum* herb. The most pronounced inhibition of alfa-glucosidase was observed for gemin A and quercetin-3-*O* glucuronide. According to Quideau [38], *Herba Sanguisorbae* contains sanguiin H-6 and H-11, while herb *Herba Geum japonicum* is rich in dimeric ellagitannins called gemin A, B, C, and both herbs have medicinal use. Tocai (Motoc) et al. [24] identified in *Sanguisorba* ellagitannins, derivatives of ellagic acid, quercetin glycoside and quercetin glucuronide, kaempferol glucuronide, hydroxycinnamic acids, flavan-3-ols, and, only in flowers, cyanidin glucoside and cyanidin malonyl glucoside.

Comparing the herbs with berries, we can see that the herbs are richer sources of ellagitannins. In the fruit of strawberry (*Fragaria ananassa* Duchesne), raspberry (*Rubus ideaus*), and blackberry (*Rubus fructicosus*), there are ETs in the following quantities: 800 mg/kg, 3000 mg/kg, and 4000 mg/kg, with a predominant share of agrimoniin (in strawberry), and a mixture of lambertianin C and sanguiin H-6 (in raspberries and blackberries) [39,40,41]. The use of fruit for the production of juices, mostly, is associated with the production of by-products in the form of a pomace, with a high proportion of seeds rich in dietary fiber, fatty acids, and a mixture of condensed tannins and ET. The amount of dried pomace of strawberry and raspberry, calculated as fresh fruits, is 2% and 6%, while the number of dried seeds, 1% and 5%. Dried raspberry pomace and raspberry seeds contain 3.9 and 3.2% of ETs with a predominant share of lambertianin C or sanguiin H-6 [40]. In turn, dried strawberry and strawberry seeds contain 1.6 and 1.0% of ETs, with a predominant share of agrimoniin [42]. Industrial strawberry and raspberry pomace, despite the high content of ETs, is not a satisfactory source of extracts rich in ETs to be used as a dietary supplement. That is because of the high health risk that the possible dietary supplements may cause due to the presence of pesticide residues, which accumulate in pomace, especially in the flesh [43,44]. Raspberry (*Rubus ideaus*), blackberry *(Rubus fructicosus*), and wild strawberry (*Fragaria vesca*) belong to medicinal plants, from which, especially from wild forms, dried plant materials are obtained: leaves *Folium Rubi idaei* and *Folium Rubi fruticosii*, and fruits *Fructus Rubi idaei* and *Rubi fruticos*. Dried blackberry and raspberry leaves collected during the flowering period contain almost 5% of tannins, as analyzed by the traditional method using leather dust. In phytotherapy, the therapeutic properties of blackberry and raspberry extracts are attributed to pirogal (ellagic) tannins. The use of blackberry leaves and fruits against bleeding, diarrhea, colds, and cough is known from the earliest times.

The structure and biological properties of phytochemicals of berries, pomegranates, nuts, and medicinal plants, especially hairy agrimony (*Agrimonia pilosa* L.) containing ETs is of interest to many world scientific centers, and in many cases, transformation of ETs into ellagic acid and urolithins occurring in the digestive tract of humans and experimental animals is the most studied subject [6,7,12,29,45,46,47,48,49,50,51,52,53]. Research on ETs of berries such as strawberry, raspberry, and blackberry [41,42,54,55,56] showed that they have a beneficial effect on the lipid profile of the blood by reducing total cholesterol, LDL-cholesterol, and triglycerides, preventing the adverse effects of a diet that is too rich in sugars [57] or saturated fatty acids [58]. In the research by Juśkiewicz et al. [59,60], it was proven that strawberry ET reduced both the level of lipids and glucose in the blood. The effect of strawberry ET on lowering blood glucose was also demonstrated in Jarosławska et al. [61]. In turn, in Kosmala et al. [41], it was shown that the blackberry fiber preparation containing ETs is less effective in reducing blood lipids compared to the raspberry fiber preparation [42]. There are also many indications that ETs have anti-inflammatory activity [14,62]. ETs in the host’s gastrointestinal tract modify the properties of the microbiota, including enzymatic activity, thus affecting the profile of secreted short-chain fatty acids [42]; increasing the proportion of propionic acid positively affects the metabolism of lipids in the liver and increasing the proportion of butyric acid has a potential anti-cancer effect in the large intestine. Much depends on the ET structure, mainly on their degree of polymerization [58].

Large ET molecules cannot be absorbed directly into the bloodstream. They must be hydrolyzed, mainly by enzymes of the microbiota living in the large intestine; the host enzymes play a much smaller role here. ETs are hydrolyzed into urolithins, as follows: the ellagic acid or HHDP (Hexahydoxydiphenoyl) is released from the ET, then it is converted into poly-hydroxylated dibenzopyranone, and then 3,4,8,9,10-pentahydroxy-6H-dibenzo[b,d]pyran-6-one (Uro-M5). Sequential dehydroxylation leads to tetrahydroxy- (Uro-M6 and Uro-D), trihydroxy- (Uro-M7 and Uro-C), dihydroxy- (Uro-A and IsoUro-A), and monohydroxy- (Uro-B) dibenzopyranones. Only these metabolites can be absorbed and changed under the influence of phase II enzymes in the form of glucuronides and sulfate conjugates [6,45,46,47,48,49,50,51,63]. Depending on the species and individual characteristics, the resulting ellagitannins may vary [64]. People can be divided into low and high urolithin producers [55]. Furthermore, some people will produce type A urolithins and other type B urolithins, while some individuals will not produce urolithins (UM-A, UM-B, and UM-0) [53,63]. The importance of the emerging ET metabolite, urolithins, still requires a lot of research. It is known that they show a weaker antioxidant effect than ETs [65]; they can activate phase II enzymes responsible for antioxidant activity and detoxification (thioredoxin reductase-1 or glutathione peroxidases) [1]; they have anti-inflammatory properties [13]; and thanks to the structural similarity to estrogens, they have anti-estrogenic/estrogenic properties [15] and anti-cancer activity [7,66]. ETs that have not been absorbed and have not undergone hydrolysis may, in turn, affect the intestinal microbiota [13]. In our previous research, the ET extract obtained from strawberry pomace reinforced the prebiotic effect of fructooligosaccharides (FOS), which resulted in a decrease in the pH of the cecum digesta, and a decrease in the concentration of putrefactive fatty acids. In turn, FOS, by activating the enzyme microbiota, increased the proportion of formed ET metabolites, while the high-fat diet limited the metabolic activity of the microbiota of the large intestine [67]. It has been proven that the degree of polymerization of ETs affects their ability to reduce the level of glucose in the blood, with monomers being more effective than dimers [59]. Previously, we have proved that ellagic acid and monomeric ET are more easily metabolized to urolithins than dimeric ET [39]. In research on ETs from dimmers to heptamers obtained from rosebay willowherb (*Epilobium angustifolium*) flowers on ruminal fermentation in vitro, it has been proven that oligomeric ETs decreased gas production and total volatile fatty acid concentration proportionally to their degree of oligomerization [68]. One of the propositions to deliver oligomeric ETs to the body was a construction of double emulsion gels loaded with ETs (in this case, *Rubus chingii Hu*) to enhance their gastrointestinal digestion stability, the delivery efficiency, and absorption [69].

As shown above, ETs have a significantly beneficial impact on human health. Analyzed medicinal plants (*Sanguisorba*, *Geum*, and *Agrimony*) are several times a richer source of ET than berries. The comparison with berry fruits such as *Fragaria ananassa*, *Rubus idaeus*, and *Rubus fruticosus* was intended to emphasize the shared phytochemical background within the Rosaceae family, particularly regarding the presence of ETs and other hydrolysable tannins. However, the analogy between the studied herbs and these fruits extends beyond ETs. The investigated medicinal herbs (*Sanguisorba officinalis* L., *Agrimonia procera* Wallr., and *Geum urbanum* L.) and the berry fruits contain other similar classes of polyphenolic compounds, including flavonols (e.g., quercetin and kaempferol derivatives), phenolic acids (ellagic acid), and proanthocyanidins.

Moreover, as the herbs are resistant to fungal diseases and pathogens, they do not need pesticides for protection. They are tasty and can be the basis of dishes and salads, being also a source of dietary fiber and vitamin C. They can also be used to prepare hot beverages. More research is needed to confirm whether herbs and their ETs have effects similar to the beneficial properties of berries in animal model studies and dietary studies involving human volunteers.

## 4. Materials and Methods

Medicinal plants (*Sanguisorba officinalis* L. (great burnet), *Geum urbanum* L. (wood avens), and *Agrimonia procera* Wallr. (fragrant agrimony)) were collected in the early stage of development (May). Analysis. Nutrients [70]: Protein content of fruit products—AOAC 920.152; Fat (Ether Extract of Plants) AOAC 930.09; dry matter—ash content of fruit—AOAC 940.26, total dietary fiber in foods (TDF) AOAC 985.29. Phenolics: Extraction of polyphenols was carried out in two steps: first with 70% methanol containing 0.1% HCOOH (three times in ultrasound bath), followed by 70% acetone containing 0.1% HCOOH (three times in ultrasound bath). The methanol and acetone extracts were combined before UHPLC analysis. UHPLC analysis: Dionex Ultimate 3000 UHPLC with DAD and MS detection (Q Exactive Orbitrap, Thermo Fisher Scientific, Waltham, MA, USA). Column Luna C18 (2) 100 Å (250 × 4.6 mm 5 µm) (Phenomenex, Torrance, CA, USA) with the same precolumn (4 × 3 mm), temperature 35 °C, detection at 250 nm, 280 nm, 320 nm, and 360 nm, flow 1 mL/min, inject volume 20 µL. Phase A: 1% (*v*/*v*) formic acid in water, phase B acetonitrile/methanol/water, *v*/*v*/*v* (83/20/17). Gradient: 0–6 min, 5% phase B; 6–36 min, 5–28% phase B; 36–73 min, 28–73% phase B; 48–54 min, 73% phase B; 54–60 min, 73–5% phase B; 60–70 min 5% phase B. MS detector parameters were as follows: Xcalibur™ 4.3 software (Thermo). Mass spectrometer Orbitrap with H-ESI probe in negative mode. Source parameters were evaporator temperature 500 °C, ion spraying voltage 4 kV, capillary temperature 400 °C, flow rate of protective gas and auxiliary gas 75 and 20 units. Full MS (*m*/*z* 200–2000) or full MS/dd-MS2 scanning modes were applied. Standards used were agrimoniin (for ET), quercetin glucoside, quercetin rhamnoside, quercetin-3-O-galactoside, apigenin glucoside, ellagic acid, chlorogenic acid, luteolin-7-O-glucuronide, kaemferol-3-O-β-d-(6″-E-p-coumaryl)-glucopyranoside, kaempferol-3-O-glucoside.

Flavan-3-ols: flavan-3-ols were determined by phloroglucinolysis. Briefly, to 20 mg of dried samples, 0.8 mL of reagent (75 g/L phloroglucinol and 15 g/L vitamin C in methanol) was added. Phloroglucinolysis was started by a 0.4 mL addition of 0.2 mol/L hydrochloric acid in anhydrous methanol. Incubation was 30 min at 50 °C with constant shaking. The reaction was stopped by placing the samples in an ice bath and adding 0.6 mL of 40 mmol/L sodium acetate. Separation was made on Gemini 5µ C18 110 Å (250 × 4.6 mm, 5 μm). Column temperature was 30 °C, Smartline chromatogram with P2800 UV–Vis detection and RF-10XL fluorescence detection (Shimadzu, Tokyo, Japan). Phase A was 2.5% aqueous solution of acetic acid, phase B 80% acetonitrile in water (*v*/*v*). Gradient 0–10 min, 4–5% phase B; 10–27 min, 7–30% phase B; 27–29 min, 30–70% phase B; 29–34 min, 70% phase B; 34–35 min, 70–4% phase B; 35–40 min, 4% phase B.

Vitamin C—extraction with 2.5% meta-phosphoric acid, detection by Knauer Smartline (Berlin, Germany) with DAD, column Phenomenex Gemini 5u C18 110A 250 × 4.60 mm; 5 μm (Phenomenex, Torrance, CA, USA). HPLC phase—KH_2_PO_4_ pH 2.5, temperature 25 °C, flow rate: 1 mL/min, detection at 254 nm.

Statistical analysis—one-way ANOVA with a statistical significance of *p* ≤ 0.05 was used (Statistica 12, Statsoft, Kraków, Poland).

## 5. Conclusions

Most of the research on *Sanguisorba officinalis* L., *Geum urbanum* L., and *Agrimonia procera* Wallr. concentrates on the medicinal properties of these herbs. A lot of research concentrates on rhizomes, which are concentrated sources of ellagitannins, but are difficult to eat in their direct form. In this research, we concentrated on plants in the early stage of development. They are a several times richer source of ETs than berries. The comparison with berry fruits such as *Fragaria ananassa*, *Rubus idaeus*, and *Rubus fruticosus* highlighted the shared phytochemical profile within the Rosaceae family, particularly the presence of ETs and other hydrolysable tannins. However, the similarity between the examined herbs and these fruits extends beyond ET. The medicinal herbs (*Sanguisorba officinalis* L., *Agrimonia procera* Wallr., and *Geum urbanum* L.) and the berry fruits contain other comparable classes of polyphenolic compounds, including flavonols, phenolic acids, and proanthocyanidins. 

Due to their resistance to fungal diseases and pathogens, selected medicinal plants are free from pesticides. Herbs of *Sanguisorba*, *Geum*, and *Agrimony* are tasty and can be the basis of dishes and salads. All the herbs can also be used to prepare hot beverages. A limitation may be a bitter taste of fragrant agrimony. The bitter taste is connected to the high tannin content in the fragrant agrimony herb, but in the early stage of plant development (which was the stage investigated in the research), the bitter taste is not intense and does not limit the nutritional use of the plant. Moreover, a bitter taste can be sought after. It does not interfere with arugula, and hops are specifically added to beer for this purpose.

Fresh avens, burnet, and agrimony greens are also a good source of dietary fiber and vitamin C and can be used in a diet with potential beneficial effects on the human body. More research in model animal systems may contribute to understanding the impact of these on the fermentation functions of the large intestine and on biochemical parameters of blood, and whether the active ingredients have an impact on reducing the risk of civilization diseases, taking into account some components of metabolic syndrome.

Sensory analysis and consumer tests should also be conducted to confirm the food appeal of the studied herbs.

## Figures and Tables

**Table 1:** Composition of herbs, g/100 g fresh matter.

	DM	TDF	IDF	SDF	Ash	Protein	Fat	Carbs	Vit C	Phenolics
*G. burnet*	19.7 ± 0.1 b	6.5 ± 0.1 c	5.7 ± 0.1 c	0.7 ± 0.1 b	1.8 ± 0.0 c	4.0 ± 0.4 a	0.7 ± 0.0 a	3.8 ± 0.0 a	0.1 ± 0.0	3.0 ± 0.2 b
*W. avens*	18.7 ± 0.1 c	8.2 ± 0.1 b	7.7 ± 0.0 b	0.5 ± 0.1 c	2.2 ± 0.1 a	2.8 ± 0.0 b	0.4 ± 0.0 b	3.0 ± 0.0 c	0.1 ± 0.0	2.1 ± 0.1 c
*F. agrimony*	23.9 ± 0.0 a	11.1 ± 0.1 a	10.2 ± 0.1 a	0.9 ± 0.1 a	2.0 ± 0.1 b	3.6 ± 0.4 a	0.4 ± 0.0 b	3.4 ± 0.0 b	0.1 ± 0.0	3.4 ± 0.0 a

Values in columns marked with different letters are statistically different at *p* ≤ 0.05. G. burnet—great burnet *Sanguisorba officinalis* L., W. avens—wood avens *Geum urbanum* L., F. agrimony—fragrant agrimony *Agrimonia procera* Wallr., DM—dry matter, TDF—total dietary fiber, IDF—insoluble dietary fiber, SDF—soluble dietary fiber, Carbs—metabolized carbohydrates, Vit C—vitamin C.

**Table 2:** Phenolic composition of herbs, mg/100 g dry matter.

mg/100 g DM	Great Burnet	Wood Avens	Fragrant Agrimony
Flavan-3-ols	0 ± 0 c	73 ± 10 b	3909 ± 25 a
Ellagitannins	12,947 ± 90 a	9689 ± 302 b	5972 ± 250 c
Hydroxycinnamic acids	129 ± 20 b	739 ± 35 a	125 ± 32 b
Ellagic acid	622 ± 30 a	84 ± 20 b	0 ± 0 c
Flavonols	835 ± 14 a	54 ± 14 b	789 ± 60 a
SUM	14,554 ± 122 a	10,857 ± 132 b	13,933 ± 101 a

Values in rows marked with different letters are statistically different at *p* ≤ 0.05. Great burnet *Sanguisorba officinalis* L., Wood avens *Geum urbanum* L., Fragrant agrimony *Agrimonia procera* Wallr.

**Table 3:** *Sanguisorba officinalis* L. phenolics identification.

Tentative Name	RT	MS/MS, *m*/*z*	[M–H]^−^	References
Neochlorogenic acid	9.1	191.06	353.09	[19] standard
Chlorogenic acid	10.8	191.06	353.09	[19] standard
Galloyl-bis-HHDP glucose isomer	11.8	467.03; 783.06	935.07 z = 1	[30,31]
Lambertianin C isomer	29.2	783.06; 934.06; 1401.59,	1869.45 z = 3	[30,31]
Sanguiin H6	30.3	309.06; 934.06	1870.13 z = 1	[19,24,31,32]
Lambertianin C isomer	31.4	783.06; 934.06; 1567.13	1869.13 z = 1	[30,31]
Galloyl-bis-HHDP glucose isomer	32.0	783.06; 433.04; 301.06	935.07 z = 1	[24,30,31]
Ellagic acid pentoside isomer	35.9	301.06	433.04	[24,31]
Ellagic acid	38.2		301.06	[24,31] standard
Ellagic acid pentoside isomer	36.7	301.06	433.04	[24,31]
Quercetin glucoside	39.0	301.04	463.03	[19] standard
Quercetin glucuronide	39.5	301.04	477.06	[24,31] standard
Quercetin rhamnoside	42.0	301.05	447.09	standard

**Table 4:** *Geum urbanum* L. phenolics identification.

Tentative Name	RT	MS/MS, *m*/*z*	[M–H]^−^	References
Galloyl-bis-HHDP glucose isomer	11.8	467.01; 783.06	935.07 z = 1	[1,30,31]
p-Coumaric acid	20.5		341.09 z = 1	
p-Coumaric acid	21.1		341.09 z = 1	
o-caffeoylquinic acid	24.7		353.06 z = 1	
Gemin A	32.9	1764.65; 1569.15, 937.10; 783.07; 613.05; 465.07; 301.06	1871.01 z = 1	[33]
Ellagic acid pentoside isomer	35.6	301.06	433.04 z = 1	[33]
Quercetin glucoside	39.0	301.04	463.03 z = 1	[33] standard
Quercetin glucuronide	39.5	301.04	477.06 z = 1	[33] standard
Kaempferol-3-O-glucoside	41.7	285.01	463.03 z = 1	[33] standard

**Table 5:** *Agrimonia procera* Wallr, phenolics identification.

Tentative Name	RT	MS/MS, *m*/*z*	[M-H]^−^	References
β-peduculagin	7.8	613.01; 481.01; 301.06	783.07 z = 1	[22]
α-peduculagin	9.4	613.01; 481.01; 301.06	783.07 z = 1	[22]
o-caffeoylquinic acid	17.3		353.06 z = 1	[22]
Epicatechin	21.5		289.07 z = 1	[22]
Catechin	23.8		289.07 z = 1	[22]
Agrimoniin	36.02	1647.58; 1567.15; 1265.14; 1085.08; 935.08; 738.07; 633.07; 613.05; 301.06	[934.08] z = 2	[22] standard
Quercetin-3-O-rhamnoglucoside	37.7	301.05	609.15 z = 1	[22] standard
Quercetin-3-O-galactoside	38.0	301.04	463.09 z = 1	[22] standard
Kaempferol-3-O-glucoside	39.3	285.04	447.04 z = 1	[22] standard
Luteolin-7-O-glucuronide	39.8	285.02	461.07 z = 1	[22] standard
Apigenin 7-O-glucuronide	42.8	269.01	445.08 z = 1	[22] standard
Kaemferol-3-O-β-d-(6′′-E-p-coumaryl)-glucopyranoside	45.9	285.01	593.13 z = 1	[22] standard

## Data Availability

The data presented in this study are available on request from the corresponding author.
